# Clinical Significance of Frizzled Homolog 3 Protein in Colorectal Cancer Patients

**DOI:** 10.1371/journal.pone.0079481

**Published:** 2013-11-08

**Authors:** Sze Chuen Cesar Wong, Catherine Wan He, Charles Ming Lok Chan, Amanda Kit Ching Chan, Heong Ting Wong, Moon Tong Cheung, Lewis Lai Yin Luk, Thomas Chi Chuen Au, Man Kin Chiu, Brigette Buig Yue Ma, Anthony Tak Cheung Chan

**Affiliations:** 1 Department of Health Technology and Informatics, Faculty of Health and Social Sciences, Hong Kong Polytechnic University, Hong Kong Special Administrative Region, China; 2 State Key Laboratory in Oncology in South China, Sir Y K Pao Centre for Cancer, Department of Clinical Oncology, Hong Kong Cancer Institute and Prince of Wales Hospital, the Chinese University of Hong Kong, Hong Kong Special Administrative Region, China; 3 Department of Pathology, Queen Elizabeth Hospital, Hong Kong Special Administrative Region, China; 4 Department of Pathology, Kiang Wu Hospital, Macau Special Administrative Region, China; 5 Department of Surgery, Queen Elizabeth Hospital, Hong Kong Special Administrative Region, China; National Cancer Center, Japan

## Abstract

Frizzled homolog 3 receptor was up-regulated in several gastrointestinal cancers such as esophageal and gastric cancers. Moreover, frizzled homolog 3 has recently reported to be expressed in colorectal adenoma specimens. In the present study, we investigated the clinical significance of frizzled homolog 3 protein in colorectal cancer patients. Using immunocytochemical staining, frizzled homolog 3 expression was examined in 186 colorectal cancer specimens, 79 colorectal adenoma specimens, 133 colorectal polyp specimens, 127 colorectal cancer specimens with lymph node and/or distant metastasis, 310 specimens of various non-colorectal cancer metastatic carcinomas and 40 specimens with simultaneous occurrence of colorectal cancer, colorectal adenoma and colorectal polyp. Statistical analysis was used to correlate frizzled homolog 3 protein expression to the clinicohistopathological factors, recurrence/metastasis and survival after follow-up for 42 months in colorectal cancer patients. Frizzled homolog 3 protein was expressed in 100% colorectal cancer specimens, 89% colorectal adenoma specimens, 75% colorectal polyp specimens and 69% normal colorectal epithelial tissues. Moreover, frizzled homolog 3 immunocytochemical scores were highly correlated with colorectal cancer progression. Furthermore, frizzled homolog 3 was expressed in a comparatively lower percentage of metastatic hepatocellular carcinoma and metastatic renal clear cell carcinoma with focal and very weak staining than other metastatic tumor types. On the other hand, the frizzled homolog 3 immunocytochemical scores of colorectal adenomas with synchronous colorectal carcinomas were significantly higher than those of pure colorectal adenomas. Statistical analysis showed that frizzled homolog 3 immunocytochemical scores were associated with Dukes stage and lymph node status. Finally, stratified groups of colorectal cancer patients had significant differences in their recurrence/metastasis and survival. In conclusion, the present large-scale study has clearly showed that frizzled homolog 3 protein can generate clinically important information for colorectal cancer patients.

## Introduction

Colorectal cancer (CRC) is the third most frequently diagnosed cancer worldwide, accounting for more than 1 million cases and 600,000 deaths every year [1]. The development of CRC is a multi-step process and this disease can be managed if detected in its early stage when it is mostly symptomless [2-4]. Despite considerable improvements in the detection, diagnosis and treatment of CRC in the last few decades, the long-term prognosis of metastatic CRC patients remains poor [5,6]. Clinical evidence shows that the current staging criteria which are based on clinical and pathologic findings, although recently revised, do not reflect the variation in prognosis and survival for metastatic CRC [7]. Therefore, an accurate prognostic marker which 1) determines the biological nature and behavior of CRC, and 2) reflects the survival differences and clinical experiences, would be of utmost importance to optimize individual treatment of CRC patients.

Wnt factors comprise a large family of secreted glycoproteins that participate in embryonic development by regulating tissue patterning, organogenesis, specification of the body plan and promote tissue homeostasis in the adult [8]. At the cellular level, Wnt factors control proliferation, differentiation, survival, motility and polarity [9]. Tight control of Wnt signaling is crucial for the correct orchestration of development and aberrant constitutive activation of the Wnt signaling pathway will lead to uncontrolled cell proliferation, growth and survival, therefore promoting the development of cancer [9]. Frizzled homolog protein (FZD) is a seven-pass transmembrane type receptor and ten members (FZD1-FZD10) have been identified in humans [10]. Wnt is a ligand of FZDs and consists of 19 family genes in humans [11]. The binding of Wnt ligands to their cell-surface receptors transduces intracellular signals through either canonical or non-canonical Wnt-signaling pathway. In the canonical Wnt signaling pathway, Wnts bind to a complex of FZD and low-density lipoprotein-related receptors 5 and 6 [12-14]. The resultant signals prevent β-catenin phosphorylation by a multiprotein complex composed of adenomatous polyposis coli, glycogen synthase kinase 3β, casin kinase 1 and axins, and promote the stabilization of cytosolic β-catenin which some of it will translocate into the nucleus [12-14]. The nuclear β-catenin will associate with T-cell factor /lymphocyte enhancer transcription factors to activate target genes involved in cell survival, proliferation or invasion [12-14]. The non-canonical Wnt signaling pathway consists of the Wnt/Ca^2+^ pathway and Wnt/c-Jun N-terminal kinase (planar cell polarity) pathway and Wnts bind to a complex comprised of FZD, receptor tyrosine kinase-like orphan receptor 2/receptor related to tyrosine kinase for activation of downstream signaling [12-14]. In Wnt/Ca^2+^ pathway, Wnt activates intracellular Ca^2+^ signaling, as well as Ca^2+^ - dependent protein kinases, such as protein kinase C and calmodulin dependent protein kinase II [12-14]. In the Wnt/JNK pathway, receptor stimulation activates Dishevelled, which in turn activates the Rho family of GTPases such as RhoA and Rac [12-14]. RhoA stimulates c-Jun expression through phosphorylation of c-Jun by Rho associated kinase [12-14]. In summary, FZD plays a crucial role in both canonical and non-canonical pathways.

Human *FZD3* was mapped to chromosome 8p21 [10,15]. *FZD3* mRNA is expressed in various normal tissues (skeletal muscle, kidney, pancreas, cerebellum and cerebral cortex) [10] whereas it is up-regulated in several cancers (esophageal carcinoma, lung squamous cell carcinoma, primary acute and lymphoblastic leukemia, myeloma, lymphoma and Ewing sarcoma) [16-19]. A recent report has shown that *FZD3* mRNA was expressed in a small cohort of sporadic adenoma and familial adenomatous polyposis adenoma tissues [20]. The significance of it is not known yet and therefore it will be interesting to examine the expression of FZD3 protein in a large cohort of primary and metastatic CRC samples and colorectal adenoma (CAD) samples. The information obtained will be very useful for us to examine the prognostic potential of FZD3 protein in patients with CRC. In addition, the expression of FZD3 protein in other types of metastatic cancers was also examined in order to explore whether FZD3 protein can be used as an adjunct diagnostic marker for metastatic CRC. 

## Materials and Methods

### Ethics Statement

The study was conducted according to the principles expressed in the Declaration of Helsinki. Written informed consent was obtained from all patients or the next of kin for use of those samples in research. Since patient consent was not possible for archival tissues as some patients were deceased at the time of study, the ethics committee specifically waived the need for consent from those patients. The study was approved by the Clinical Research Ethics Committee of the Queen Elizabeth Hospital, Hong Kong Special Administrative Region (HKSAR).

### Tissue Specimens

Seven cohorts of formalin fixed, paraffin-embedded (FFPE) specimens were recruited: 1) CRC with Dukes classification system stage A (43), stage B (48), stage C (63), stage D (32), total: 186, 2) CAD with mild dysplasia (25), moderate dysplasia (28), severe dysplasia (26), total: 79, 3) colorectal polyp which is specifically defined as non-adenomatous or non-neoplastic polyps which consist of juvenile (35), hyperplastic (40), Peutz-Jeghers (28) and inflammatory polyps (30), total: 133, 4) one FFPE block with lymph node metastasis from each Dukes C specimen, total: 63, 5) one FFPE block each with lymph node and distant metastasis from each Dukes D specimen, total: 64, 6) three hundred and ten non-CRC metastatic carcinomas which the primary tumor was respectively hepatocellular carcinoma (HCC) (23), renal clear cell carcinoma (RCCC) (25), squamous cell carcinoma (SCC1) (30), lung adenocarcinoma (LAC) (25), lung non-small cell carcinoma (LNSCC) (27), papillary thyroid carcinoma (PTC) (24), breast carcinoma (BC) (28), ovary clear cell carcinoma (OCCC) (30), cervical non-small cell carcinoma (CNSCC) (20), fallopian tube serous adenocarcinoma (FTSAC) (22), transitional cell carcinoma (TCC) (23), small cell carcinoma (SCC2) (15) and carcinoma with neuroendocrine differentiation (CND) (18), 7) specimens from forty CRC patients (10 Dukes A, 10 Dukes B, 12 Dukes C, 8 Dukes D) with simultaneous occurrence of CRC, adenoma and polyp (120), were retrieved from the archives (2005-2008) of the Anatomical Pathology and Cytology Laboratory, Department of Pathology, Queen Elizabeth Hospital, HKSAR, for FZD3 immunocytochemical (ICC) staining. 

Most cases of the colorectal adenocarcinomas in the first cohort were moderately differentiated (78%), whereas the rest were either well differentiated (5%) or poorly differentiated (17%). The sex distribution of adenocarcinoma patients was 58% male and 42% female. The age ranged from 42 to 89 years old. Of the 79 adenoma specimens examined in cohort 2, 59% was tubular, 35% was tubulovillous, and 6% was villous adenomas. The percentages of mild, moderate, and severe dysplasia in adenomas were 32, 35 and 33, respectively. The sex distribution of adenoma patients was 53% male and 47% female. The age ranged from 28 to 85 years old. The polyps evaluated in cohort 3 were juvenile (26%), hyperplastic (30%), Peutz-Jeghers (21%), and inflammatory polyps (23%). Of the 133 polyps, the sex distribution was 51% male, 49% female and the age ranged from 23 to 76 years old. The clinical information of the patients with simultaneous occurrence of CRC, adenomas and polyps in the seventh cohort were moderately differentiated (75%), well differentiated (7%) or poorly differentiated (18%). The sex distribution of adenocarcinoma patients was 60% male and 40% female. The age ranged from 45 to 87 years old.

### Antibody

Monoclonal mouse anti-human FZD3 antibody (1:100 dilution, clone 169310, MAB1001, R&D Systems, Inc., Minneapolis, USA) was used.

### ICC staining and evaluation

Serial tissue sections (4 μm thick) were cut and antigen retrieval was performed using Bond Epitope Retrieval Solution 2 on the Bond-max automated immunostainer (Vision BioSystems, Mount Waverley, Australia) at 100°C for 25 minutes. Staining was performed according to a standard protocol in the immunostainer. Polymer detection system was selected to avoid the problem of nonspecific endogenous biotin staining. Esophageal carcinoma was used as a positive control [16] which was mounted on every test slide and negative controls were performed by replacing the antibody with tris buffered saline. In order to measure the FZD3 expression in each patient specimen accurately, a maximum number of target cells with the staining intensity which reflects the general expression pattern is the main criteria for scoring. The stained slides were evaluated in at least 5 fields with the highest number of target cells under light microscope at X400 magnification by 2 independent observers without knowledge of clinical outcomes and in the case of disagreement, consensus was reached after thorough discussion and slides examination using a multi-headed microscope. Approximately 250 cells would be counted in each field and therefore at least 1,250 cells would be counted for each patient specimen. All slides were scored semi-quantitatively and expressed as an ICC score by multiplying the “percentage of positive cells” and the “staining intensity”, as described previously [21]. Staining intensity was scored as follows: 0 = negative; 1 = weak; 2 = moderate; 3 = strong; and 4 = very strong. The ICC score ranged from 0 to 400. The scoring of percentage and staining intensity was targeted to 1) CRC cells, 2) CAD cells and 3) adjacent normal epithelial cells. Membranous staining should be the expected result. However, our experience showed that ICC staining using diaminobenzidine as chromogen would produce a thickened membranous staining which was similar to cytoplasmic staining. Besides, the antibody used in this study had the best performance among the antibodies which had been tested. 

### Statistical analysis

The difference in ICC scores of FZD3 protein among CRC, CAD and adjacent normal colorectal epithelial cells was studied using non-parametric Kruskal-Wallis test. In addition, non-parametric Spearman rank correlation test was used to examine whether FZD3 ICC scores were correlated with CRC progression and Dukes classification stages. Mann Whitney U test was used to examine whether the ICC scores of CAD with synchronous colorectal carcinomas had significant difference from those of pure CAD. For correlation to clinical parameters, multivariate regression was used to analyze whether FZD3 ICC scores were correlated with the clinico-histopathological factors of the patients and chi-square test was used to examine the association between FZD3 ICC scores and recurrent or metastatic CRC. The overall survival was defined as that from the date of the operation to the date of death due to cancer. The Kaplan-Meier method was used to determine the probability of survival and log-rank test was used to compare the survival between the groups and Cox proportional hazards model was applied to determine the independent prognostic factors of survival. (Statistical Package for the Social Sciences Version 16.0 software for Windows, IBM SPSS statistics, Chicago, IL., USA). A *P* value < 0.05 was considered to be statistically significant in all analyses. All *P* values are two-tailed. 

## Results

### ICC staining

As mentioned in the ICC staining and evaluation section, a thickened membranous staining which resembled cytoplasmic staining was detected. Among the 955 slides that we have examined, inter-individual variation was found only in 16 slides (1.7%) and consensus was reached after discussion using a multi-headed microscope. 

**Figure 1:**
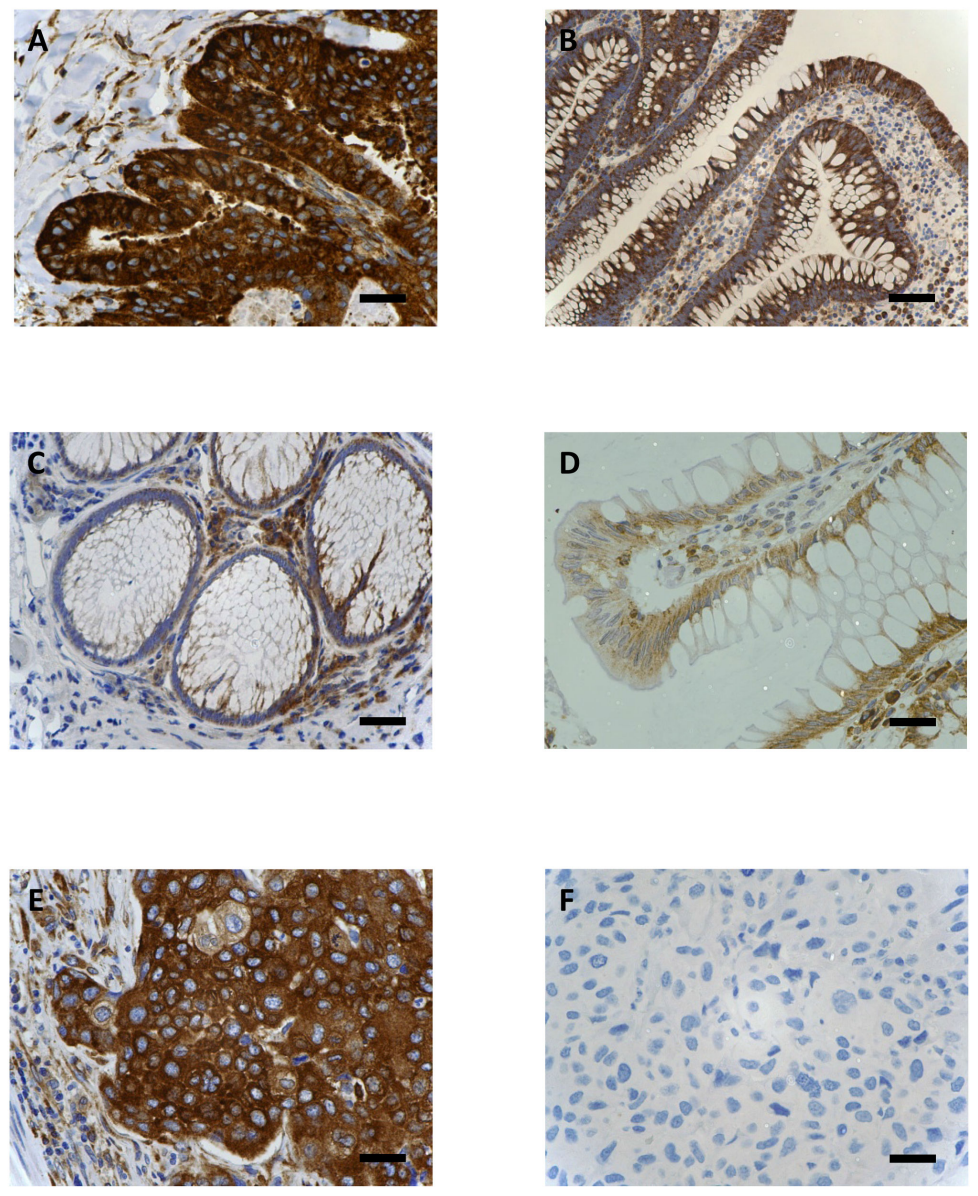
ICC staining of FZD3 in (A) CRC, (B), CAD, (C) colorectal polyp, (D) normal colorectal tissues, (E) positive control and (F) negative control (Original magnification, X400, scale bar = 100 µm).

**Figure 2:**
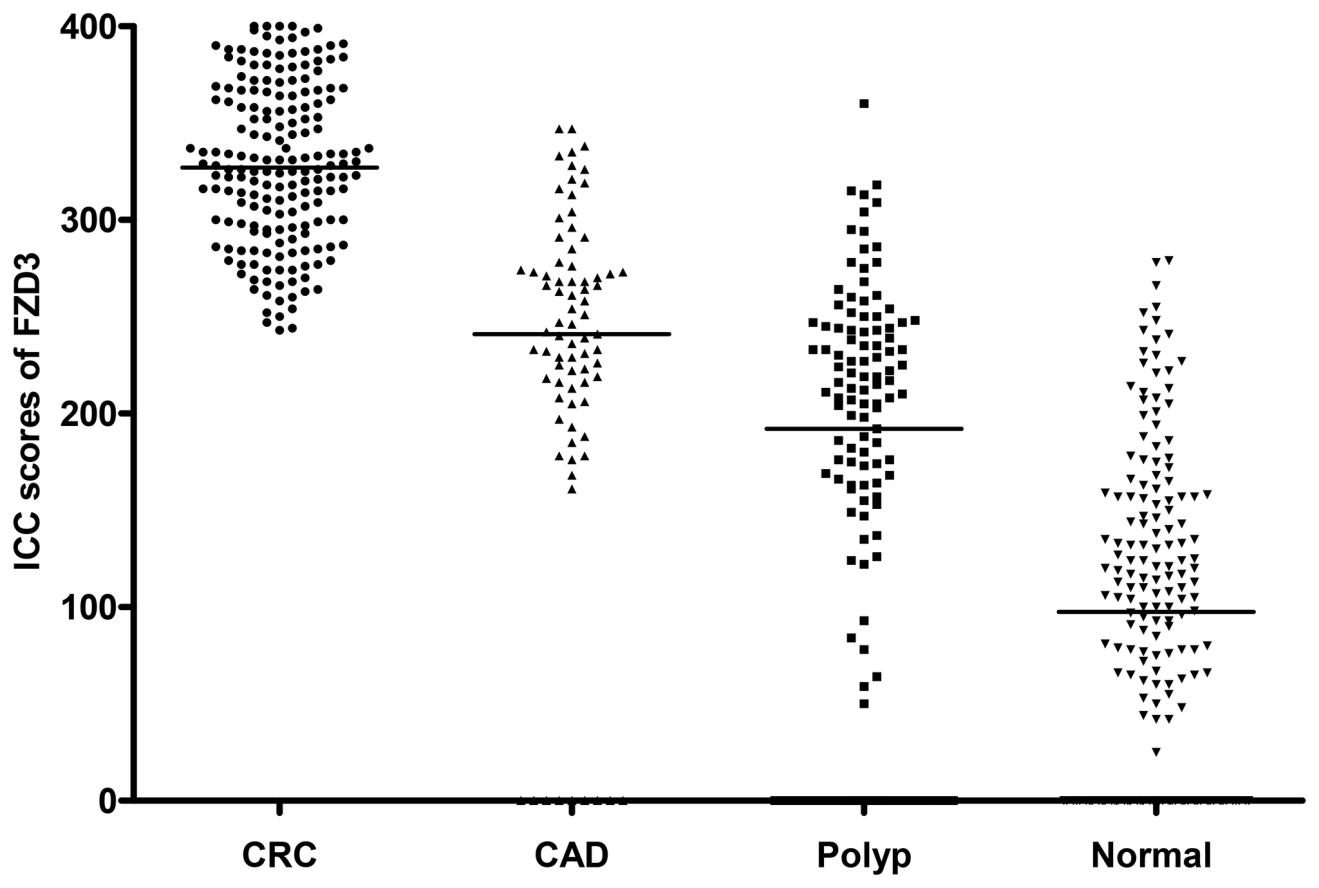
ICC scores of FZD3 in (A) CRC, (B) CAD, (C) colorectal polyp and (D) normal colorectal tissues.

**Figure 3:**
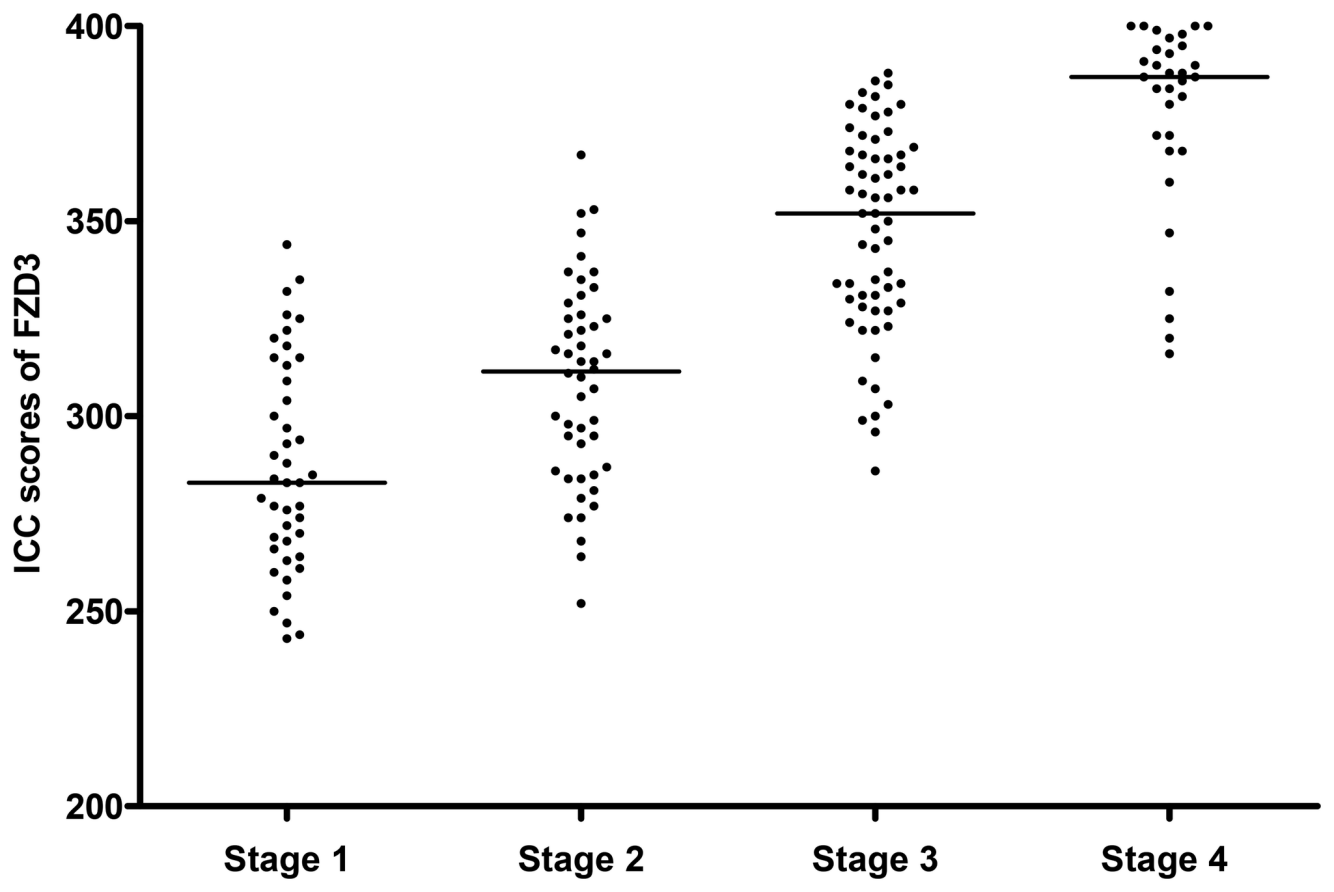
ICC scores of FZD3 in various Dukes stage of patients with CRC.

**Figure 4:**
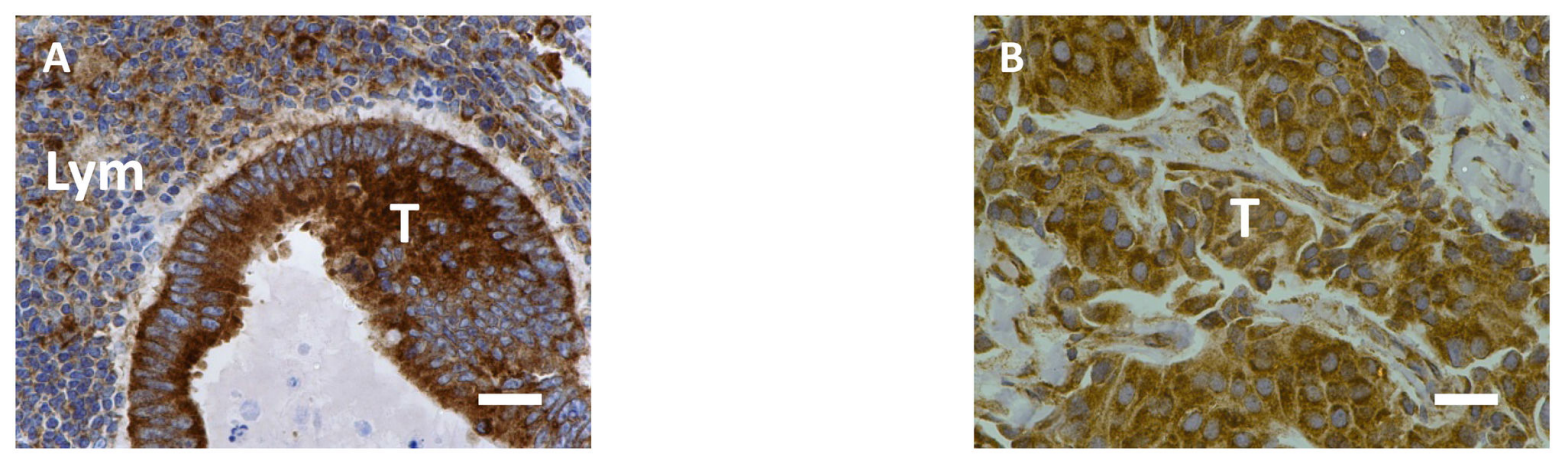
ICC staining of FZD3 in (A) a lymph node with CRC metastasis from a Dukes 3 patient and (B) an ovary with CRC metastasis from a Dukes 4 patient (T = tumor, Lym = lymphoid cells, original magnification, X400, scale bar = 100 µm).

**Figure 5:**
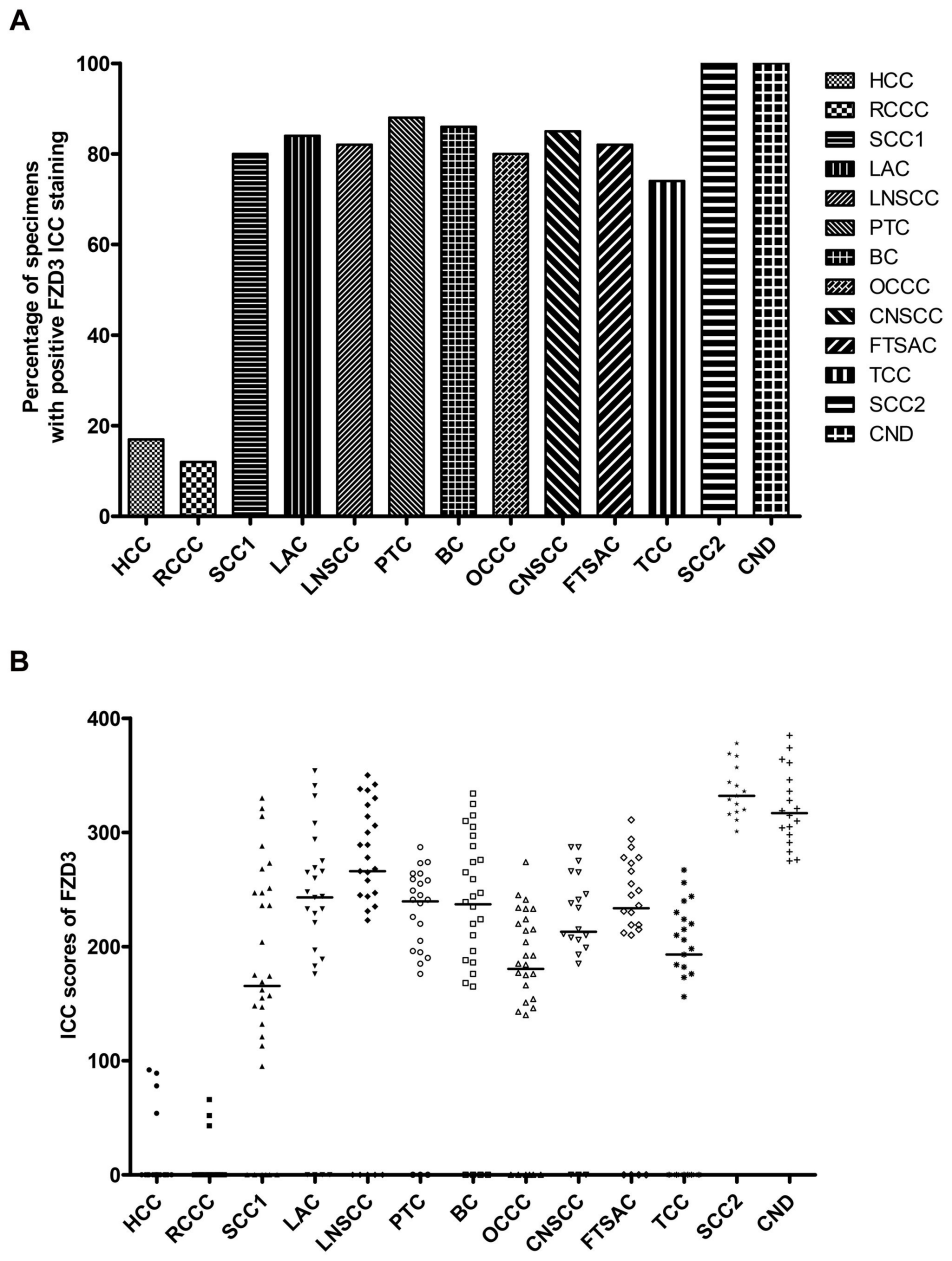
FZD3 expression in various non-CRC metastatic carcinomas. (A) Percentage of various non-CRC metastatic carcinomas with FZD3 ICC staining and (B) ICC scores of FZD3 in various non-CRC metastatic carcinomas. (HCC = hepatocellular carcinoma, RCCC = renal clear cell carcinoma, SCC1 = squamous cell carcinoma, LAC = lung adenocarcinoma, LNSCC = lung non-small cell carcinoma, PTC = papillary thyroid carcinoma, BC = breast carcinoma, OCCC = ovary clear cell carcinoma, CNSCC = cervical non-small cell carcinoma, FTSAC = fallopian tube serous adenocarcinoma, TCC = transitional cell carcinoma, SCC2 = small cell carcinoma and CND = carcinoma with neuroendocrine differentiation).

**Figure 6:**
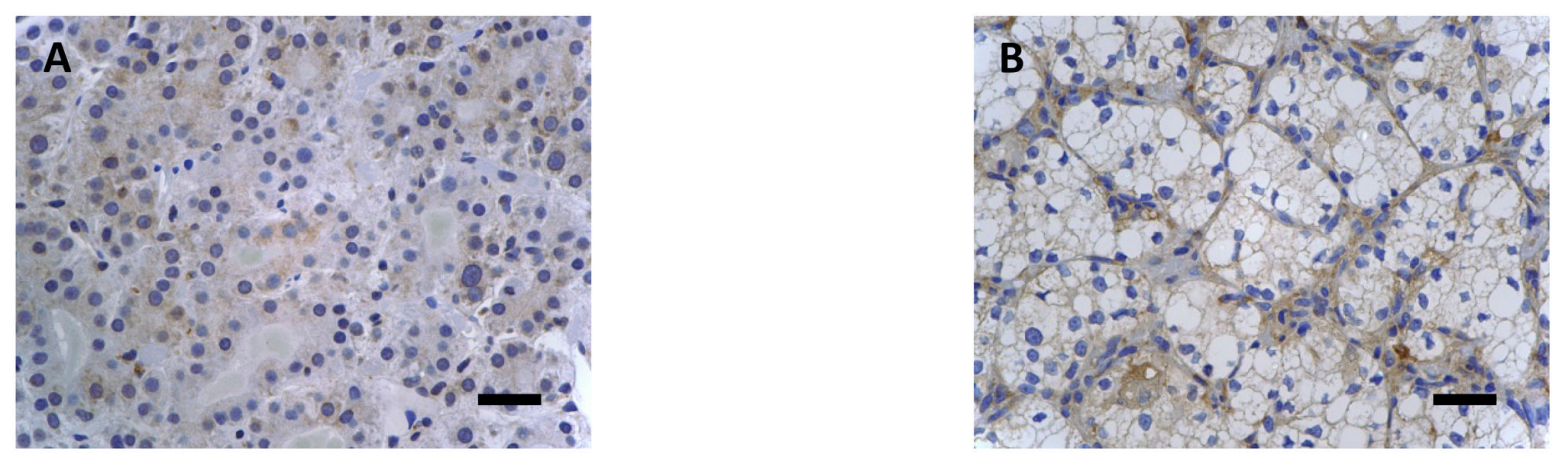
ICC staining of FZD3 in (A) a metastatic hepatocellular carcinoma and (B) a metastatic renal clear cell carcinoma (Original magnification, X400, scale bar = 100 µm).

ICC staining showed that FZD3 protein was expressed in 100% (186/186) of CRC specimens, 89% (70/79) of CAD specimens, 75% (100/133) of colorectal polyp specimens and 69% (129/186) of normal colorectal epithelial tissues adjacent to CRC tissues. A representative photomicrograph of each specimen type in the first cohort was shown in Figure 1. Positive control of esophageal carcinoma showed intense positive cytoplasmic staining whereas negative control did not have any ICC staining (Figures 1E and 1F). Detailed analysis showed that the immunoreactivity of FZD3 protein, as shown by the ICC scores, had significant difference among CRC, CAD, colorectal polyp and normal colorectal epithelial tissues (Figure 2, *P* < 0.0001, Kruskal-Wallis test) and the range and median of the ICC scores in various specimen types were shown in Table 1. FZD3 ICC scores were highly correlated with the stages of progression of CRC (*P* < 0.0001, Spearman rank correlation test). Moreover, the ICC scores of FZD3 protein in CRC specimens was significantly correlated with Dukes stage (Figure 3, *P* < 0.0001, Spearman rank correlation test). Furthermore, our findings showed that FZD3 protein was expressed in 100% metastatic CRC cells in lymph nodes or/and distant organs from Dukes 3 and 4 specimens, respectively (Figure 4). In line with those results, we continued to examine whether FZD3 can be used as an adjunct diagnostic marker for metastatic CRC by performing FZD3 ICC staining in 310 non-CRC metastatic carcinoma specimens. Results showed that FZD3 protein was expressed in 17% (4/23) of HCC, 12% (3/25) of RCCC, 80% (24/30) of SCC1, 84% (21/25) of LAC, 82% (22/27) of LNSCC, 88% (21/24) of PTC, 86% (24/28) of BC, 80% (24/30) of OCCC, 85% (17/20) of CNSCC, 82% (18/22) of FTSAC, 74% (17/23) of TCC, 100% (15/15) of SCC2 and 100% (18/18) of CND (Figures 5A and 5B). In cases with positive results, detailed examination showed that the staining was focal and very weak in HCC, RCCC when compared to those in other tumor types (Figures 5B and 6). 

**Table 1:** The range and median of the ICC scores in various specimen types.

	Colorectal cancer	Colorectal adenoma	Colorectal polyp	Adjacent normal epithelial tissue
Number of specimens	186	79	133	186
Range of ICC scores	243 to 400	0 to 347	0 to 360	0 to 279
Median ICC scores	327	241	192	97.5

**Figure 7:**
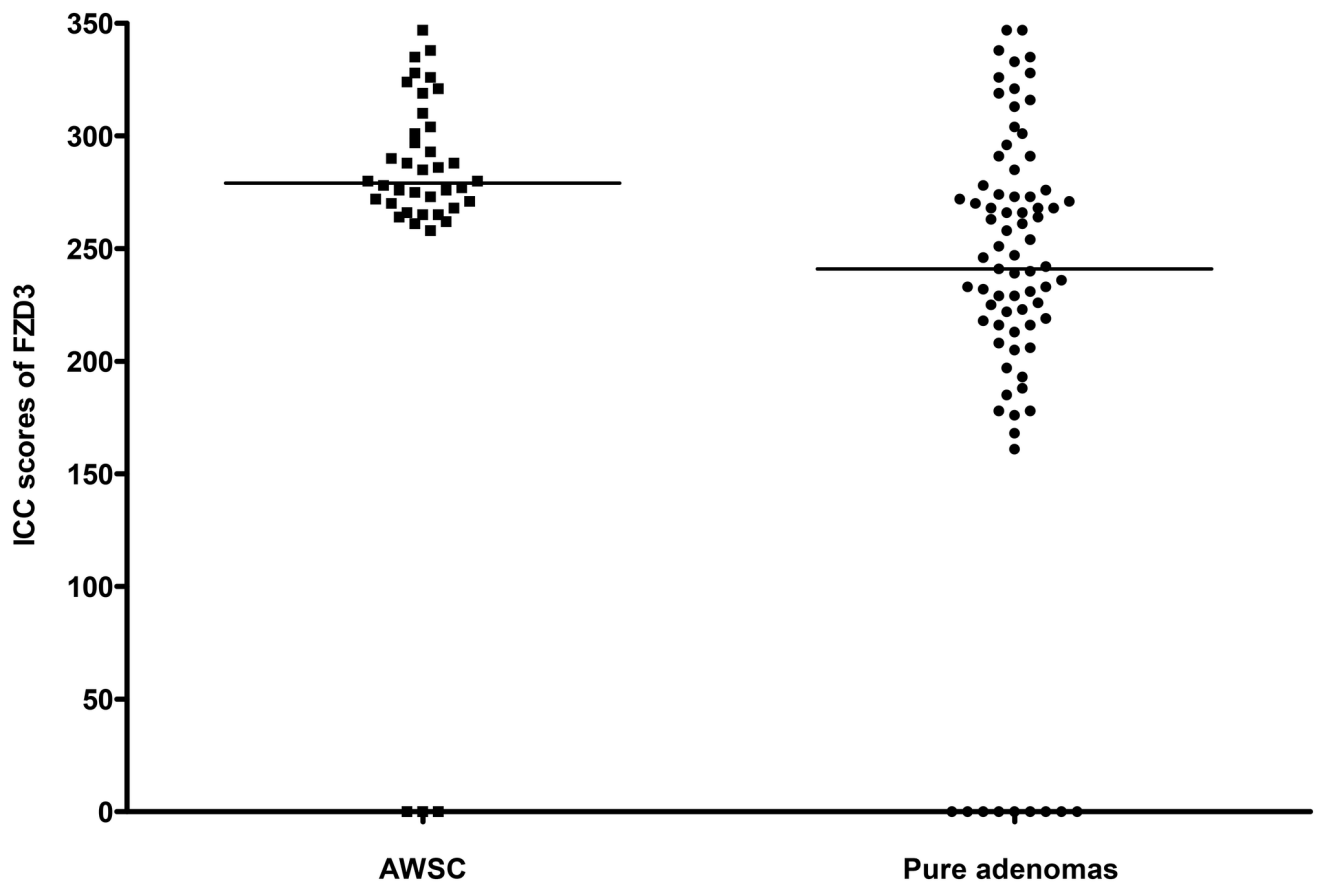
ICC scores of FZD3 in (A) adenomas with synchronous carcinomas (AWSC) and (B) pure adenomas.

Similar observations were found for specimens with simultaneous occurrence of colorectal carcinoma, CAD and colorectal polyp. ICC staining for FZD3 was observed in 100% (40/40) of CRC, 93% (37/40) of CAD, 70% (28/40) of colorectal polyp. Moreover, FZD3 ICC scores were highly correlated with the stages of progression of CRC (*P* < 0.0001, Spearman rank correlation test). Of interest, the ICC scores of CAD with synchronous colorectal carcinomas were significantly higher than those of pure CAD (*P* < 0.001, Mann Whitney U test, Figure 7). 

### Multivariate regression analysis

Multivariate regression analysis was applied to examine whether FZD3 ICC scores were correlated with the clinicohistopathological factors of the 186 Dukes A to D CRC patients. Significant associations were found with Dukes stage (*P* < 0.001) and lymph node status (*P* < 0.05) but not for age (*P* = 0.547), sex (*P* = 0.813), tumor stage (*P* = 0.284) and degree of differentiation (*P* = 0.462). 

### Recurrent or metastatic CRC

The median number of FZD3 ICC scores from the 154 Dukes A to C CRC patients was 321.5. Using this median number as the cut-off point, 27 patients with FZD3 ICC score > 321.5 and 8 patients with FZD3 ICC score ≤ 321.5 had recurrent or metastatic CRC after follow-up for 42 months and the association between FZD3 ICC scores and recurrent or metastatic CRC was highly significant (Chi-square test: χ^2^ = 13.34; *P* < 0.001). 

### Overall survival of CRC patients

**Figure 8:**
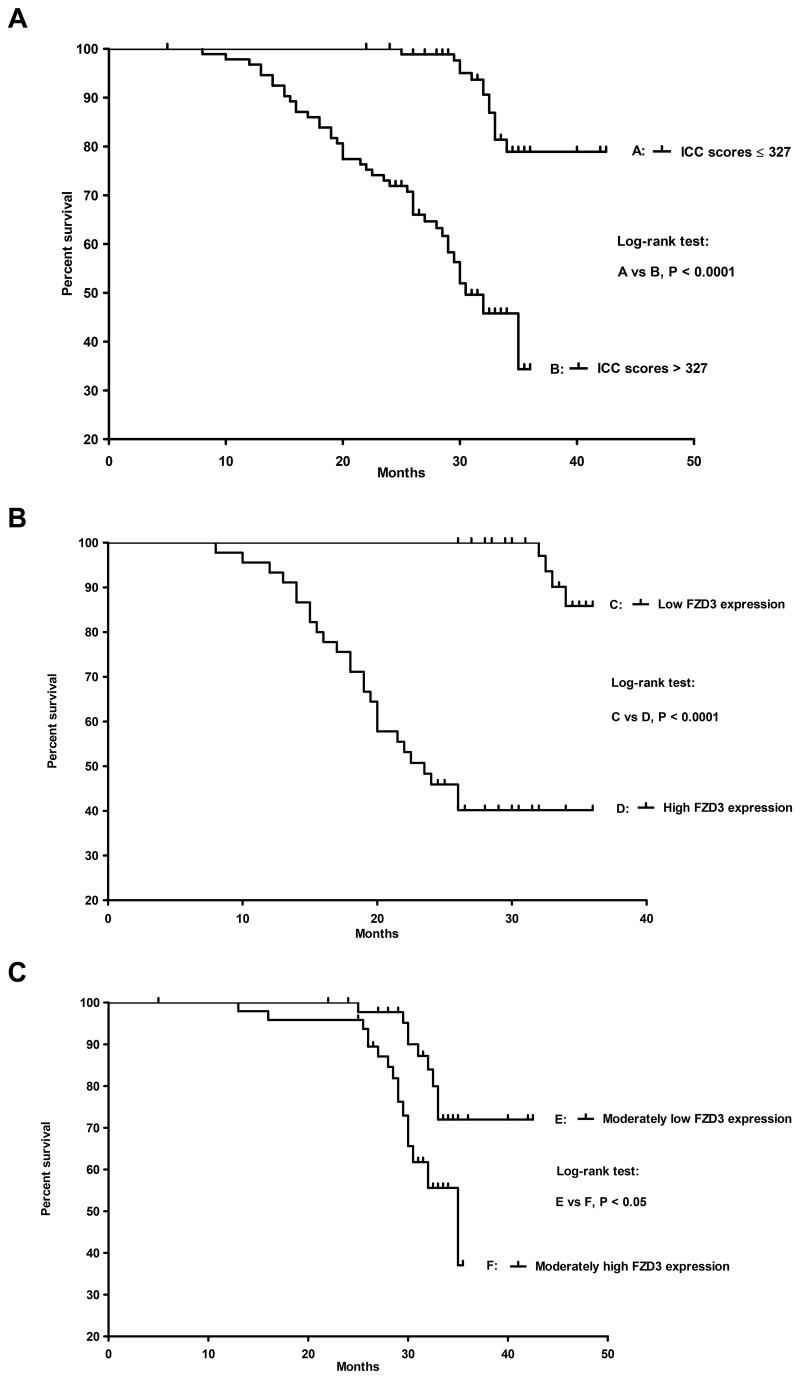
Overall survival analyses for the 1^st^ cohort of 186 Dukes A to D CRC patients (A) stratified by a median FZD3 ICC score of 327, (B) with high and low FZD3 expression and (C) with moderately high and moderately low FZD3 expression.

Overall survival curves were plotted for CRC patients with FZD3 ICC scores > 327 and those with FZD3 ICC scores ≤ 327 where 327 is the median number of FZD3 ICC scores from the 1^st^ cohort of 186 Dukes A to D CRC patients. Our results showed that the survival for those 2 groups of patients was significantly different (*P* < 0.0001, log-rank test, Figure 8A). In order to analyse the data in a more specific manner, the FZD3 ICC scores were divided into 4 groups after aligning it in descending order: 1) high FZD3 expression: highest 25% (76% to 100%) ICC scores, 2) moderately high FZD3 expression: second highest 25% (51% to 75%) ICC scores, 3) moderately low FZD3 expression: second lowest 25% (26% to 50%) ICC scores and 4) low FZD3 expression: lowest 25% (0% to 25%) ICC scores. As shown in Figures 8B and 8C, the survival between groups 1 and 4 and groups 2 and 3 were significant (*P* < 0.0001 for A vs D and *P* < 0.05 for B vs C, log-rank test). Finally, the independent prognostic factors of overall survival identified by the Cox proportional hazards regression model were found to be FZD3 ICC score (*P* = 0.016) and lymph node status (*P* = 0.037). 

## Discussion

FZD3 is a receptor for the Wnt-signaling pathway and FZD3 protein is required for the development of major fiber tracts in the rostral central nervous system [22], neural crest [23] and hair follicle development [24]. Moreover, FZD3 protein has a predominant role in Wnt-induced neurite outgrowth in Ewing sarcoma tumor cells [25] and it is highly expressed in poorly differentiated squamous cell esophageal carcinoma tissues [16]. To the best of our knowledge, this study is the first to examine the clinical significance of FZD3 protein expression in a large cohort of CRC and its pre-malignant CAD and colorectal polyp specimens. Despite the fact that FZD3 protein was expressed in 100% of CRC specimens, 89% of CAD specimens, 75% of colorectal polyp specimens and 69% adjacent normal colorectal epithelial tissues, the immunoreactivity of FZD3 had significant difference among those 4 groups of specimens. Moreover, FZD3 protein expression in CAD as shown by us matches with those of a previous study that FZD3 mRNA expressed in 79% (11/14) CAD specimens with a median fold change of 6.5 [26]. However, FZD3 mRNA was only found to have a significant up-regulation in 39% (9/23) CRC specimens in the same study [26]. The reasons account for this conflicting result can be explained by the following reasons: 1) a small number (23) of CRC specimens were recruited as compared to ours, 2) mRNA was detected whereas protein was detected in our study, 3) adjacent normal tissues were mixed with the CRC tissues as no microdissection was performed in their study. The last point is especially significant because variable degrees of adjacent normal tissues will usually be found in surgical specimens and our findings showed that FZD3 protein was expressed not only in 69% of adjacent normal colorectal epithelial tissues, but also in fibroblasts, endothelial cells, lymphocytes and histiocytes (data not shown). ICC staining of a tissue section allows us to examine the individual cell under microscope and therefore the FZD3 protein expression in tumor and normal cells can be separately quantified. In reality, the ICC staining of FZD3 protein was not only stronger in the invasive front than in the tumor center but also very intense in all metastatic lymph nodes and distant organs. This remarkable finding can show that FZD3 protein has an important role not only in early CRC carcinogenesis, but also in CRC metastatic pathway to lymph nodes and distant organs. However, focal polarized distribution of FZD3 as shown by Pourreyron et al. was not found in our cohort of specimens [27]. We continued to investigate the potential of FZD3 protein as an adjunct diagnostic marker in metastatic CRC by performing ICC staining in various types of non-CRC metastatic carcinomas. Our results are very interesting because ICC staining for FZD3 was positive to various extents in all metastatic carcinomas. However, the positive staining in only 17% of HCC and 12% of RCCC shows that FZD3 protein may not be so essential in the metastatic process of those 2 carcinomas (Figures 5A and 5B). In summary, FZD3 ICC staining may be helpful to exclude those 2 carcinomas upon pathological diagnosis because the staining was focal and very weak in those FZD3 staining positive HCC and RCCC cases when compared to those in other tumor types. On the other hand, FZD3 protein was expressed in metastatic carcinomas which include SCC1, LAC, LNSCC, PTC, BC, OCCC, CNSCC, FTSAC, TCC, SCC2 and CND. Those novel results can show that FZD3 protein may be involved in the metastatic process of those carcinomas. In fact, the strong staining of FZD3 protein in all cases of CND and SCC2 is not surprising because FZD3 protein is well known to be involved in central nervous system development [22,23,28]. In line with those results, we are currently examine the FZD3 protein expression in their respective primary tumors for those 11 types of metastatic non-CRC carcinomas which have high level of FZD3 protein expression in order to have a more understanding on the role of FZD3 protein in various carcinogenesis pathways.

 Another interesting finding from this study is that the median ICC score of the CAD associated with synchronous colorectal carcinomas is much higher than that of the group consisting of pure CAD, suggesting that a high FZD3 protein expression in CAD may signify a higher malignancy potential. 

 The ultimate goal of this study is to explore the clinical significance of FZD3 protein in patients with CRC. As currently there is scanty pathologically used prognostic marker for CRC and therefore it is invaluable to investigate the prognostic potential of FZD3 protein which is demonstrated in this study by the significant correlation of FZD3 ICC scores to Dukes stage, lymph node status, recurrence or metastasis. Moreover, a long-term follow-up of those CRC patients showed that patient with a high FZD3 ICC score may have a less chance of survival. Actually, the specimens with FZD3 ICC scores > 327 were mainly of stages III and IV patients whereas those with FZD3 ICC scores ≤ 327 were mostly of stages I and II patients. This may be one explanation why stages III and IV patients have a greater risk of recurrence, metastasis and shorter survival than stages I and II patients. A more detailed analysis of the data by classifying it into 4 groups based on their level of expression can provide us more information on the prognostic significance of FZD3 protein in CRC patients. The significant difference in survival between high and low expression groups is expected whereas the marginal significant difference (*P* < 0.05) in survival between moderately high and moderately low expression groups provides a solid evidence that FZD3 protein has important prognostic power. Taken all those findings together, FZD3 ICC staining can potentially be a valuable tool for the prognosis of CRC. In order to validate those findings, a large multi-centre trial with a long-term follow-up on examining its prognostic significance is now being prepared. Human Wnt5A, Wnt5B and Wnt11 are representative non-canonical Wnts transducing Wnt/planar cell polarity signals through FZD3 receptors [10,29-31]. A recent study has shown that Wnt response pathways were re-organized in early colorectal carcinogenesis as the negative regulator of Wnt signaling pathway, naked cuticle homologue 1 induction was accompanied by up-regulation of the β-catenin-independent FZD3 receptor in CAD and CRC [26]. Moreover, FZD3 did not respond to stabilization of β-catenin *in vitro* and required a Wnt ligand signal [26]. Therefore, FZD3 may provide an indication of the degree of Wnt ligand signaling in the colorectal tumor. In fact, a small cohort of specimens (20) with strong and weak positive FZD3 were recruited and a direct comparison of FZD3 immunostaining to those of beta-catenin were performed and it was found that there was no correlation between FZD3 and beta-catenin immunostaining (data not shown). This finding can strengthen the role of FZD3 in non-canonical Wnt signaling pathway. Together with the results from this study, the expression of FZD3 protein in various clinical stages of CRC can further demonstrate the clinical significance of FZD3 in patients with CRC. Our next step is to examine the underlying molecular mechanisms that regulate FZD3 expression and the direct transcriptional target genes of FZD3 using specific knockdown of FZD3 by small interfering RNA in CRC cell lines because targeted therapy using molecular markers is an important goal for improving the outcomes of patients with CRC. Furthermore, the detection of FZD3 protein in patient’s serum using enzyme-linked immunosorbent assay will also be explored in order to assess whether serum FZD3 protein can be used as a non-invasive marker. However, a major limitation of this study is that we do not have the clinical information to assess the performance of FZD3 protein by comparing its expression to conventional CRC marker such as serum carcinoembryonic antigen. 

 In summary, FZD3 protein expression as shown by ICC staining is closely correlated with colorectal carcinogenesis and progression, and can potentially serve as a new prognostic marker. Moreover, FZD3 ICC staining may be helpful to exclude HCC and RCCC in metastatic sites. 
